# Mendelian randomisation to uncover causal associations between conformation, metabolism, and production as potential exposure to reproduction in German Holstein dairy cattle

**DOI:** 10.1186/s12711-025-00950-w

**Published:** 2025-02-25

**Authors:** Leopold Schwarz, Johannes Heise, Zengting Liu, Jörn Bennewitz, Georg Thaller, Jens Tetens

**Affiliations:** 1 Department of Animal Sciences, Georg-August-University, 37077, Göttingen, Germany; 2 Vereinigte Informationssysteme Tierhaltung w.V. (VIT), 27283 Verden, Germany; 3 Institute of Animal Science, University of Hohenheim, 70599 Stuttgart, Germany; 4 Institute of Animal Breeding and Husbandry, Christian-Albrechts-University, 24118 Kiel, Germany

## Abstract

**Background:**

Reproduction is vital to welfare, health, and economics in animal husbandry and breeding. Health and reproduction are increasingly being considered because of the observed genetic correlations between reproduction, health, conformation, and performance traits in dairy cattle. Understanding the detailed genetic architecture underlying these traits would represent a major step in comprehending their interplay. Identifying known, putative or novel associations in genomics could improve animal health, welfare, and performance while allowing further adjustments in animal breeding.

**Results:**

We conducted genome-wide association studies for 25 different traits belonging to four different complexes, namely reproduction (n = 13), conformation (n = 6), production (n = 3), and metabolism (n = 3), using a cohort of over 235,000 dairy cows. As a result, we identified genome-wide significant signals for all the studied traits. The obtained summary statistics collected served as the input for a Mendelian randomisation approach (GSMR) to infer causal associations between putative exposure and reproduction traits. The study considered conformation, production, and metabolism as exposure and reproduction as outcome. A range of 139 to 252 genome-wide significant SNPs per combination were identified as instrumental variables (IVs). Out of 156 trait combinations, 135 demonstrated statistically significant effects, thereby enabling the identification of the responsible IVs. Combinations of traits related to metabolism (38 out of 39), conformation (68 out of 78), or production (29 out of 39) were found to have significant effects on reproduction. These relationships were partially non-linear. Moreover, a separate variance component estimation supported these findings, strongly correlating with the GSMR results and offering suggestions for improvement. Downstream analyses of selected representative traits per complex resulted in identifying and investigating potential physiological mechanisms. Notably, we identified both trait-specific SNPs and genes that appeared to influence specific traits per complex, as well as more general SNPs that were common between exposure and outcome traits.

**Conclusions:**

Our study confirms the known genetic associations between reproduction traits and the three complexes tested. It provides new insights into causality, indicating a non-linear relationship between conformation and reproduction. In addition, the downstream analyses have identified several clustered genes that may mediate this association.

**Supplementary Information:**

The online version contains supplementary material available at 10.1186/s12711-025-00950-w.

## Introduction

Female fertility is of fundamental importance in dairy cattle. Despite this, over the decades, a decrease in reproductive performance has been observed [1, 2], while production traits like milk kg (MKG), fat kg (FKG) or protein kg (PKG) have improved and been the central focus of breeding programmes in dairy production systems [3]. Various factors can contribute to these circumstances, including an extremely low heritability for reproductive performance compared to production traits, a negative genetic correlation between these trait pairs, and management deficiencies [4–6]. To consider these factors and improve functional traits, the genetic correlation between the traits is crucial [7, 8] and remains a persistent challenge in dairy cattle breeding programmes [9]. The modifications needed for enhancement are of further interest to fulfil society’s increasing awareness of animal welfare and the ongoing changes in husbandry conditions, such as climate change [10, 11]. Moreover, genetic correlation can arise for distinctive reasons, including but not limited to linkage between causal variants or forms of pleiotropy [12, 13], i.e., more than one trait is affected by a genetic variant.

Furthermore, it is important to differentiate between genetic correlation and causation and identify the underlying causal mechanisms [12–14]. The extent and direction of genetic correlation may vary depending on the specific traits under consideration [9, 15]. Nevertheless, a detailed and direct inference about causality or causal associations from genetic correlations alone is not feasible [13, 16, 17]. It is necessary to move beyond global correlation to gain detailed genomic correlation and causality analysis and determine the connection between quantitative traits in dairy cattle breeding [18, 19].

Several established approaches and methods can be used to reveal potential causal associations between traits [14]. Randomised controlled trials (RCTs) are generally considered the gold standard for causality testing of possible exposure (e.g. treatment) against outcomes (e.g. disease) [20]. Nevertheless, RCTs are restricted in their ability to ascertain the causal relationships between quantitative traits due to the control challenges for potential confounding factors [21]. Moreover, the small sample size and controlled experimental conditions may not accurately reflect the conditions present on commercial dairy farms. It is important to note that only observational data is available, which can limit the reliability of the findings. One way to address this problem is to use single nucleotide polymorphisms (SNPs) as instrumental variables (IVs) for achieving randomisation, a method known as the Mendelian randomisation (MR) approach [12, 22, 23]. This method allows for the inference of potential causal association effects between two traits. The design of the method defines the case and control groups, revealing the potential association effects between exposure and outcome.

Burgess et al. [12] provided guidelines for MR analyses and compared the various available methods. In general, three key assumptions must be fulfilled by the IVs [24, 25]: (1) the IV is associated with the exposure, (2) the IV does not affect the outcome except potentially via the exposure, and (3) the IV is not associated with the outcome due to confounding pathways. This study used summary statistics from genome-wide association studies (GWAS) and performed MR using the generalised summary-data-based Mendelian randomisation (GSMR) approach [17]. This method identified and quantified genetic effects between traits and narrowed down potential mediators for causal association effects, such as pleiotropy [17, 26].

Briefly, trait interrelationship may arise due to varying reasons, simplified here into three major categories: (1) real (biological) pleiotropy, indicating one variant is affecting two traits similar to one variant is causative for both traits (2) mediated (vertical/indirect) pleiotropy, whereby one variant affects one trait through another, and (3) supposed real (horizontal/direct) pleiotropy, where causal variants for two traits are in linkage disequilibrium (LD) with a variant associated with both traits but may even fall in distinct loci [12, 27].

Notably, the second category, mediated pleiotropy, is particularly interesting, as it encompasses the genetic correlation employed to ascertain the underlying causal association [13]. Consequently, the principal objective of the GSMR method is to infer the causal association between traits mediated by the IVs [12, 17]. Moreover, this method has additional advantages, including identifying horizontal pleiotropic variants that affect both traits and their removal from further analyses, as well as approximating the causal effect of an exposure on an outcome [17, 26]. The most notable distinction between MR studies in livestock species and humans is the discrepancy in the availability of potential exposure data and the observational setting. While voluntary and self-determined treatments for dairy cattle are scarcely available, human studies commonly focus on self-determined behaviours such as smoking [22], alcohol consumption [28] or physical activities [29].

To better understand the interconnections between various trait complexes, we analysed traits related to reproduction, production, conformation, and metabolism to assess their potential influences on one another. The traits associated with the complexes of conformation, production and metabolism were selected for their putative exposures on the reproduction traits as the outcome, based on known or presumed genetic connections (e.g. body condition score and calving interval [30] or negative energy balance and hypothalamo-hypophyseal axis [31]).

Ketosis (KET), displaced abomasum (LMV) and milk fever (MIF) were used as potential indicators of metabolism. In addition to the body condition score (BCS**)**, milk type (MTY) and several overall scores for body (OBS), udder (OUS), feet and legs (OFL), or conformation (OCS) were used to outline conformation. Here, we used an approach based on GWAS summary statistics for 25 traits, including 235,164 German Holstein Friesian dairy cows. The identified IVs were then used for downstream analyses to identify potential candidate genes responsible for the detected effects. Additionally, we performed genetic restricted maximum likelihood (GREML) analyses to estimate variance components within a smaller dataset of 48,118 animals. The study combined information from GSMR and GREML to investigate the overall interrelationship between traits based on SNPs from GREML and IVs identified from GSMR.

## Material and methods

### Animals and phenotypes

Phenotypic and genotypic data were provided by the national computing centre VIT (Vereinigte Informationssysteme Tierhaltung w.V., Verden, Germany) and part of the ‘KuhVision’ dataset based on German Holstein cows, which represent the genomic pattern of the German Holstein Friesian reference population [8].

The number of animals with observational data per trait ranged from 110,629 to 192,188 (Table 1), representing 235,164 animals in total. We estimated the variance components using a subset of 48,118 animals that simultaneously had complete observations for all traits. To ensure clarity, we have employed the terms and abbreviations defined by VIT [32]. A total of 25 traits related to conformation, production, metabolism, or reproduction were analysed.

**Table 1:** Complex, trait, abbreviation and number of animals (No.) used for GWAS and subsequent GSMR analyses

Complex	Trait	Abbreviation	No.	h^2^ (SE)
Exposure				
Conformation	Body condition score	BCS	163,127	0.318 (0.007)
	Milk type	MTY	163,127	0.298 (0.007)
	Overall conformation score	OCS	163,127	0.281 (0.007)
	Overall body score	OBS	163,127	0.339 (0.007)
	Overall udder score	OUS	163,127	0.286 (0.007)
	Overall feet and legs score	OFL	163,127	0.108 (0.005)
Metabolism	Ketosis	KET	148,515	0.070 (0.004)
	Displaced abomasum	LMV	133,525	0.102 (0.005)
	Milk fever	MIF	145,802	0.068 (0.004)
Production	Milk kg	MKG	180,217	0.439 (0.007)
	Fat kg	FKG	180,217	0.372 (0.007)
	Protein kg	PKG	180,217	0.324 (0.007)
Outcome				
Reproduction	Non-return rate cows	NRc	163,663	0.063 (0.004)
	Non-return rate heifers	NRh	187,004	0.106 (0.005)
	First to last insemination cows	FSc	149,289	0.146 (0.005)
	First to last insemination heifers	FSh	176,091	0.091 (0.004)
	Calving to first insemination cow	CFc	165,212	0.109 (0.005)
	Days open cow	DOc	165,212	0.167 (0.006)
	Calving ease direct	CEd	130,495	0.095 (0.005)
	Calving ease maternal	CEm	185,153	0.097 (0.005)
	Stillbirth direct	SBd	138,592	0.103 (0.005)
	Stillbirth maternal	SBm	192,188	0.132 (0.005)
	Retained placenta	NGV	142,395	0.068 (0.004)
	Metritis/Endometritis	MET	126,842	0.054 (0.003)
	Ovary cycle disturbances	ZYS	110,629	0.070 (0.004)

h^2^ = SNP-based heritability estimates of the traits with their standard errors (SE)

The 13 reproduction traits reflect the calving-related traits with calving ease direct/maternal (CEd/CEm), endometritis/metritis (MET), retained placenta (NGV), stillbirth direct/maternal (SBd/SBm) or ovary cycle disturbances (ZYS). Additionally, they also include fertility indicators such as calving to first insemination cow (CFc), days open cow (DOc), first to last insemination cow/heifer (FSc/FSh) or non-return rate cows/heifers (NRc/NRh).

For this analysis, we used phenotypic data as deregressed proofs (DRPs) obtained from VIT’s 2021 breeding value estimation. The calculations were made using the deregression method by Jairath et al. [33], which aligned with its application described by Liu and Masuda [34]. However, it is important to highlight the informative value of the DRPs, which were corrected for environmental factors and the population mean. Therefore, they were taken from different steps of the national breeding value estimation in Germany. For example, a lower value for MET implies a higher susceptibility, while a lower value for CFc indicates a shorter period than the population mean.

### Genotyping data, quality control

The genotypes comprising 45,613 SNPs were obtained through routine genetic evaluation. The study mainly included animals imputed to the 50K level using various versions of the EuroGenomics low-density chips (Eurogenomics, Amsterdam, NL), with a minority genotyped using various versions of the Illumina 50K chips (Illumina Inc., San Diego, CA) and EuroGenomics medium-density chips (Eurogenomics, Amsterdam, NL).

The imputation procedure described in Segelke et al. was implemented [35]. To perform further analyses, we filtered the data using PLINK v1.90 [36], removing SNPs with a minor allele frequency below 1%. The genotype dataset used in the GCTA tool version 1.93.2 beta contained 44,994 SNPs on *Bos taurus* (BTA) autosomes and the X chromosome (BTAX) [37].

### Variance components and genetic correlations

Variance components and genetic correlations between traits were estimated using GREML [38] as implemented in GCTA [37]. The analysis was conducted on a subset of 48,118 animals. The trait variances were partitioned into an additive genetic and a residual component. This partition was based on the genetic relationship between the individuals using the autosomal SNPs (44,136) [38] in a univariate analysis. The SNP heritability (the proportion of phenotypic variance explained by the additive effects of common SNPs) was estimated. A bivariate GREML (biREML) analysis was conducted to assess the genetic correlations between traits, as described by Lee et al. [39].

### Genome-wide association studies

Each trait was split into two equal cohorts to accommodate the large number of animals per trait and meet the computational demand. GWAS was conducted separately for each cohort. To identify the genomic regions associated with each one, single-trait GWAS was performed for all 25 using GCTA [37] with a mixed-linear model approach (MLMA) using a single SNP regression [40], as implemented with the model below:

$${\mathbf{y}} = {\mathbf{1}}\mu + {\mathbf{x}}b + {\mathbf{g}} + {\mathbf{e}}$$

In this case, the phenotype vector represented as DRPs is denoted by $\mathbf{y}$, while $1$ is a vector of ones and *µ* signifies the mean term. The additive (fixed) effect of the individual candidate SNP being tested for association is represented by *b*, and the vector $\mathbf{x}$ stores the SNP genotype indicator variables coded as 0, 1 or 2 for all animals for one of the 44,994 SNPs on the autosomes and BTAX. The genetic relationship matrix (GRM) captures the polygenic effect, represented by $\mathbf{g}$, while the residual is represented by $\mathbf{e}$*.* To account for the kinship structure of the study population, the GRM was constructed using all autosomal SNPs (44,136). Subsequently, meta-analyses were conducted using the METAL program (version 2020-05-05) [41] to combine both cohorts per trait and obtain a joint test statistic. METAL utilises GWAS test statistics obtained from MLMA, which include estimated effects and standard errors per SNP. Furthermore, METAL considers individual cohorts’ sample sizes to combine *p-*values and convert them into merged signed Z-scores [41].

In addition to the default settings, the flag for genomic control correction was set to restrict the genomic inflation factor (lambda, λ) of every test statistic to a maximum value of one. To ensure accurate results, we conducted a genomic control parameter [42] estimation for each input file. We then applied genomic control correction to input statistics before performing a meta-analysis and creating the joint summary statistic.

The meta-analyses were performed twice for each trait [41]. First, the separate cohorts were combined to obtain a joint test statistic. Second, the obtained combined test statistic was re-processed to correct any putative genomic inflation arising from the combination. Therefore, to prevent *p-*value inflation, the λ values for genomic inflation were restricted to a maximum of one (λ ≤ *1*). Subsequently, the Z-scores obtained from METAL were transformed back to beta values using the method described by Zhu et al. [26]. The basic principle of transformation relies on b_zx_ (effect of the single SNP (z) on the trait, here exposure (x)) that could be interpreted in standard deviation (SD) units, i.e. b_zx_** =**$\frac{{\mathrm{Z}}_{\mathrm{zx}}}{\sqrt{2p(1-p)(n+{\mathrm{z}}_{\mathrm{zx}}^{2})}},$ with *p* being the allele frequency and *n* being the sample size [26].

For considering genetic variants as associated, the GWAS threshold for significance was Bonferroni-corrected on a genome-wide level to account for multiple SNP testing. Therefore, a type-I error value of α = 0.05 was used, divided by the number of tests (*p* = 1.11 * 10^−6^ [(0.05/44,994), − log_10_ (*p*) ≈ 5.95]). The Manhattan plots for graphical representation were generated using the R package ggplot2 [43] in R version 4.2.0 [44].

### Mendelian randomisation

Mendelian randomisation was performed using summary statistics from the above GWAS and the GSMR approach implemented in GCTA [17]. This method extends the summary-data-based MR (SMR) method [26]. It integrates the estimates of SNP effects and the distinction between causality; in this case, the influence of the SNP on the outcome is mediated by the exposure. Furthermore, it incorporates horizontal pleiotropy; in this case, the SNP has a different impact on exposure and outcome, thus accounting for LD structure and variance in both [17].

Zhu et al. provide a comprehensive explanation of the method [17]. However, briefly, the following filter settings were used: the GWAS threshold *p-*value was set to 1.11 * 10^−6^ after applying Bonferroni correction. The clumping *r*^*2*^ threshold was set to 0.05, with a minimum of ten genome-wide significant and quasi-independent SNPs used as the default for analysis. The fundamental concept is that if an exposure (x) affects an outcome (y), any instrumental variables (IVs, SNPs, z) that are causally associated with x will also affect y. Moreover, the effect of x on y (b_xy_) is expected to be identical for any SNPs without pleiotropy [17].

In this study, the MR estimate of the GSMR method, which indicates the causal effect of a specific exposure on a given outcome trait, is calculated for the individual variant (*i*) as b_xy(*i*)_ = $\frac{{\mathrm{b}}_{\mathrm{zy}(i)}}{{\mathrm{b}}_{\mathrm{zx}(i)}}$. For each combination of individual exposure against the individual outcome, the overall b_xy_ equals the sum of all the single IV effects estimated, following a normal distribution given ~ *N(*b_xy_*,* **V***)*. In this context, **V** represents the variance–covariance matrix of b_xy_, which includes the LD correlation between the IVs [17]. Using the HEIDI (heterogeneity in dependent instruments) outlier filtering [17], we crucially removed horizontal pleiotropic variants to avoid any possible violation of the MR assumptions outlined above (e.g. [25]). The fundamental concept was to test every IV for a significant deviation (*d*_*i*_) of the individual b_xy(*i*)_ from a target SNP that had a strong association with the exposure tested (b_xy(top)_).

Interestingly, the capacity to identify heterogeneity is enhanced as the correlation between the SNP and the exposure in question strengthens. However, simply selecting the most exposure-associated SNP is not feasible as there is a possibility it may have a markedly strong pleiotropic effect [17]. Therefore, to increase robustness, the distribution of b_xy_ is ordered as a function of − log_10_(*p-*value) for b_zx,_ and the SNP with the strongest association is then selected in the third quintile of the distribution itself. Given the approximation of *d*_*i*_ = b_xy(*i*)_ − b_xy(top)_ and var(*d*_*i*_) = var(b_xy(*i*)_  − b_xy(top)_), considering the LD among IVs and filtering for LD that was not removed by the clumping filter beforehand [17].

Each potential exposure trait was tested against all reproduction-associated traits as separate outcomes. For every trait combination, we obtained the instrumental variables (SNP_GSMR_) that represented them, the estimated mediated effect of the exposure on the outcome (b_xy_), both overall and per individual IV, as well as the corresponding *p-*value (*p*_GSMR_) after HEIDI filtering for the removal of pleiotropic SNPs [17]. To correct for the multiple testing on 13 different reproduction traits jointly against each single potential exposure trait and to determine the significance threshold for each combination (single exposure vs. single outcome), we used the remaining number of SNPs after applying the GWAS threshold and LD-clumping (= SNP_INDEX_) for correction. As a result, we used a threshold of *p*_GSMR_ < (0.05/SNP_INDEX_) for significance.

### Downstream analysis

The study used the Ensembl Variant Predictor (VEP) release 94 [45] with markers identified by the GSMR method and the results for SNP_GSMR_ per trait combination as the data input. The *Bos taurus* genome (assembly ARS-UCD1.2) was screened for putative genes within a 1000 base pair (bp) range downstream and upstream of each identified marker. Only known genes with confirmed symbols approved by the gene nomenclature [46] were considered. As a result of the GSMR analysis, four traits from three complexes were selected: BCS and OBS for conformation, MKG for production, and KET for metabolism.

For the conformation traits, we chose a direct trait and a linear score trait to compare. Finally, the R package VennDiagram [47] was used to generate a Venn diagram showing shared candidate genes between the four selected traits.

## Results

### Variance components and genetic correlations

Our results show that heritability estimates were low for traits representing reproduction, metabolism, and partly conformation (e.g. OFL h^2^ = 0.108, Table 1). Regarding reproductive and metabolic traits, MET had the lowest heritability (h^2^ = 0.054) and DOc the highest (h^2^ = 0.167). Moderate estimates were obtained for the other conformation traits (like BCS h^2^ = 0.318) and for production, demonstrating even higher heritability estimates (MKG h^2^ = 0.439).

We used the biREML option to estimate genetic correlations, which involved all 44,136 autosomal SNPs in the panel. For this study, these estimates are presented at three different levels: (1) between all exposure and all outcome combinations, (2) within exposure only, and (3) within outcome traits only. The results of the exposure-outcome analysis are presented in Table 2, which includes SNP-based correlation estimates and their corresponding standard errors (SE).

**Table 2:** SNP-based genetic correlation estimates (r_G_) between exposure (vertical) and outcome traits (horizontal)

Trait	CEd	CEm	CFc	DOc	FSc	FSh	MET	NGV	NRc	NRh	SBd	SBm	ZYS
BCS	0.005 (0.028)	0.003 (0.028)	− *0.373 (0.024)*	− *0.293 (0.023)*	− 0.194 (0.024)	− 0.152 (0.028)	− 0.019 (0.033)	0.118 (0.031)	0.081 (0.031)	0.040 (0.027)	0.007 (0.027)	− 0.020 (0.026)	0.166 (0.030)
FKG	− 0.032 (0.027)	− 0.033 (0.027)	*0.283 (0.024)*	*0.288 (0.022)*	*0.259 (0.023)*	*0.215 (0.026)*	0.066 (0.032)	0.036 (0.030)	− *0.208 (0.029)*	− 0.131 (0.026)	− 0.039 (0.027)	0.020 (0.025)	− 0.140 (0.029)
KET	0.163 (0.038)	0.145 (0.038)	− 0.160 (0.037)	− *0.248 (0.033)*	− *0.264 (0.033)*	− *0.238 (0.038)*	*0.260 (0.043)*	*0.275 (0.040)*	*0.211 (0.042)*	0.119 (0.037)	0.120 (0.038)	0.088 (0.036)	*0.277 (0.040)*
OBS	− 0.167 (0.027)	0.074 (0.028)	*0.210 (0.026)*	*0.229 (0.023)*	*0.207 (0.024)*	0.128 (0.028)	− 0.165 (0.032)	− 0.101 (0.031)	− 0.159 (0.031)	− 0.095 (0.027)	− 0.089 (0.027)	0.005 (0.025)	− 0.157 (0.030)
LMV	0.192 (0.035)	0.107 (0.035)	− 0.144 (0.034)	− 0.198 (0.030)	− 0.193 (0.031)	− 0.162 (0.035)	*0.333 (0.038)*	*0.245 (0.037)*	0.134 (0.039)	0.088 (0.034)	0.152 (0.034)	0.107 (0.032)	*0.285 (0.036)*
MIF	0.108 (0.039)	0.121 (0.039)	− 0.185 (0.037)	− 0.184 (0.034)	− 0.159 (0.035)	− 0.159 (0.035)	0.190 (0.044)	0.138 (0.042)	0.063 (0.043)	0.027 (0.038)	0.088 (0.039)	0.110 (0.036)	*0.258 (0.036)*
MKG	0.010 (0.026)	− 0.011 (0.026)	*0.456 (0.022)*	*0.385 (0.020)*	*0.287 (0.022)*	*0.239 (0.025)*	0.009 (0.031)	− 0.087 (0.029)	− 0.152 (0.029)	− 0.100 (0.025)	0.007 (0.026)	0.005 (0.024)	− 0.171 (0.028)
MTY	− 0.044 (0.028)	− 0.006 (0.028)	*0.388 (0.024)*	*0.319 (0.023)*	*0.217 (0.024)*	0.154 (0.028)	− 0.030 (0.033)	− 0.111 (0.031)	− 0.090 (0.031)	− 0.049 (0.027)	− 0.026 (0.028)	0.002 (0.026)	− 0.166 (0.030)
OCS	0.013 (0.029)	0.087 (0.028)	0.021 (0.028)	0.004 (0.025)	− 0.013 (0.025)	− 0.016 (0.029)	0.067 (0.033)	− 0.005 (0.031)	0.015 (0.032)	0.005 (0.028)	0.020 (0.028)	0.050 (0.026)	0.049 (0.031)
OFL	0.136 (0.035)	0.028 (0.035)	− 0.119 (0.033)	− 0.088 (0.030)	− 0.050 (0.031)	− 0.049 (0.035)	0.139 (0.040)	0.086 (0.038)	0.025 (0.039)	0.004 (0.034)	0.135 (0.034)	0.020 (0.032)	0.146 (0.037)
OUS	0.014 (0.028)	0.034 (0.028)	− 0.088 (0.027)	− 0.110 (0.024)	− 0.126 (0.025)	− 0.150 (0.029)	0.090 (0.030)	0.050 (0.031)	0.129 (0.032)	0.079 (0.028)	0.017 (0.028)	− 0.014 (0.026)	*0.209 (0.030)*
PKG	0.035 (0.028)	− 0.009 (0.028)	*0.456 (0.023)*	*0.393 (0.021)*	*0.302 (0.023)*	*0.254 (0.027)*	0.057 (0.033)	− 0.066 (0.031)	− 0.173 (0.030)	− 0.123 (0.027)	0.024 (0.027)	0.022 (0.025)	− 0.170 (0.030)

Values for r_G_ > |0.200| are displayed in italic. The standard error (SE) for each combination is displayed in parentheses. Trait abbreviations are explained in Table 1

Overall, the genetic correlations (r_G_) varied between r_G_ =  − 0.373 ± 0.024 for BCS against CFc (BCS‑CFc) and r_G_ = 0.456 ± 0.023 for PKG‑CFc. For most combinations, r_G_ values were low, with absolute values of the estimates close to the standard errors. Moderate negative genetic correlations were found for BCS‑CFc (r_G_ =  − 0.373 ± 0.024) as well as BCS‑DOc (r_G_ =  − 0.293 ± 0.023), along with FKG‑NRc (r_G_ =  − 0.208 ± 0.029).

In contrast, moderate positive genetic correlations were found for several traits, such as FKG‑CFc (r_G_ = 0.283 ± 0.024), FKG‑DOc (r_G_ = 0.288 ± 0.022), MKG‑FSc (r_G_ = 0.287 ± 0.022) and PKG‑FSh (r_G_ = 0.254 ± 0.027).

For the exposure traits (Additional file 1, Table S1), the genetic correlations ranged from r_G_ = -0.346 ± 0.019 for BCS‑PKG to r_G_ = 0.831 ± 0.005 for MKG‑PKG. For BCS‑MTY, the genetic correlation estimation failed; however, testing another model excluding the residual covariance revealed an r_G_ close to 1 between both traits (r_G_ = − 0.988 ± 0.001).

The genetic correlations for most traits (45 out of 65 combinations) were found to be low (r_G_ < |0.200|). Within the different fields, moderate (KET‑MIF) up to high genetic correlations (MKG‑PKG) were observed with r_G_ = 0.582 ± 0.028 and r_G_ = 0.831 ± 0.005, respectively.

The outcome traits (Additional file 1, Table S2) showed a range of r_G_ values, from r_G_ = − 0.786 ± 0.015 for FSh‑NRh to r_G_ = 0.912 ± 0.006 for DOc‑FSc. Of 76 trait combinations, 31 had a low genetic correlation (r_G_ < |0.200|). The estimation for two combinations, FSc‑NRc and FSh‑NRc, also failed. However, dropping the residual covariance revealed a high genetic correlation of r_G_ = − 0.985 ± 0.002 and − 0.994 ± 0.002, respectively.

### Genome-wide association studies

For all 25 traits (12 exposure, 13 outcome), genetic variants were found that passed the genome-wide significance threshold (*p* = 1.11 * 10^ −6^). Additional file 2, Tables S3 to S4, lists all genome-wide significant variants per trait and their corresponding *p*-values. Manhattan plots for all traits not shown in this section are displayed in Additional file 3, Figures S1 to S4.

#### Production traits

Signals for all three production traits were found on BTA1, 2, 3, 5, 6, 11, 14, 15, 19, 20, 26, 27, 28, 29 and BTAX. On BTA14, a region between 1,463,676 and 2,909,929 bp contained a prominent peak for all three traits, including SNP ARS-BFGL-NGS-4939 as the most significant hit.

#### Metabolic traits

**Fig. 1:**
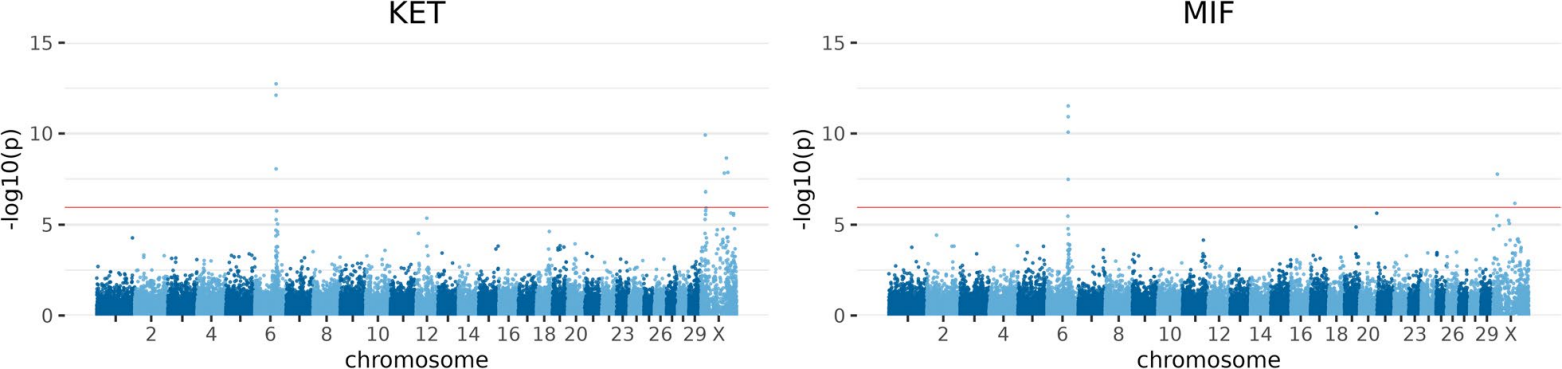
Manhattan plots for exemplary results of GWAS for metabolic traits. Ketosis (KET) and milk fever (MIF). Negative decadic logarithm of *p*-value of each SNP is shown on the y-axes, and on the x-axes, the 29 autosomes and X chromosome are shown. The red line represents the significance threshold on genome-wide level *p* = 1.11 * 10^–6^

The metabolic traits analysed were KET, LMV and MIF. Several SNPs reached the genome-wide significance threshold, with peaks on BTA6 and BTAX (KET and MIF in Fig. 1). The region between 88,592,295 and 88,891,318 bp on BTA6 contained several significant variants, three of which were common to all traits. SNP BTB-01654826 was the most significant hit across all traits in this region.

#### Conformation traits

**Fig. 2:**
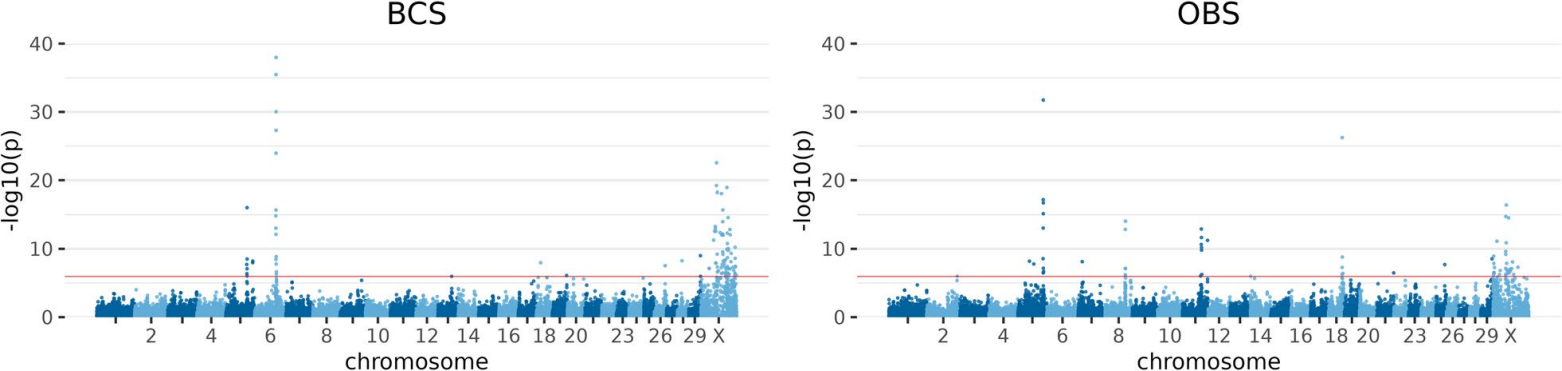
Manhattan plots for exemplary results of GWAS for conformation traits. Body condition score (BCS) and overall body score (OBS). Negative decadic logarithm of *p*-value of each SNP is shown on the y-axes, and on the x-axes, the 29 autosomes and X chromosome are shown. The red line represents the significance threshold on genome-wide level *p* = 1.11 * 10^−6^

For conformation, the traits BCS, OBS, MTY, OCS, OFL, and OUS were analysed, and peaks on BTA2, 4, 5, 6, 7, 8, 9, 11, 13, 14, 18, 19, 20, 21, 25, 26, 28, 29 and BTAX were found. The strongest signals were on BTA5, 6, 8 and 11 for BCS, OBS and MTY, including ARS-BFGL-NGS-118182 on BTA6 for BCS and MTY as well as ARS-BFGL-NGS-11105 on BTA11 for OBS. The results for BCS and OBS are displayed as examples in Fig. 2.

#### Reproduction traits

**Fig. 3:**
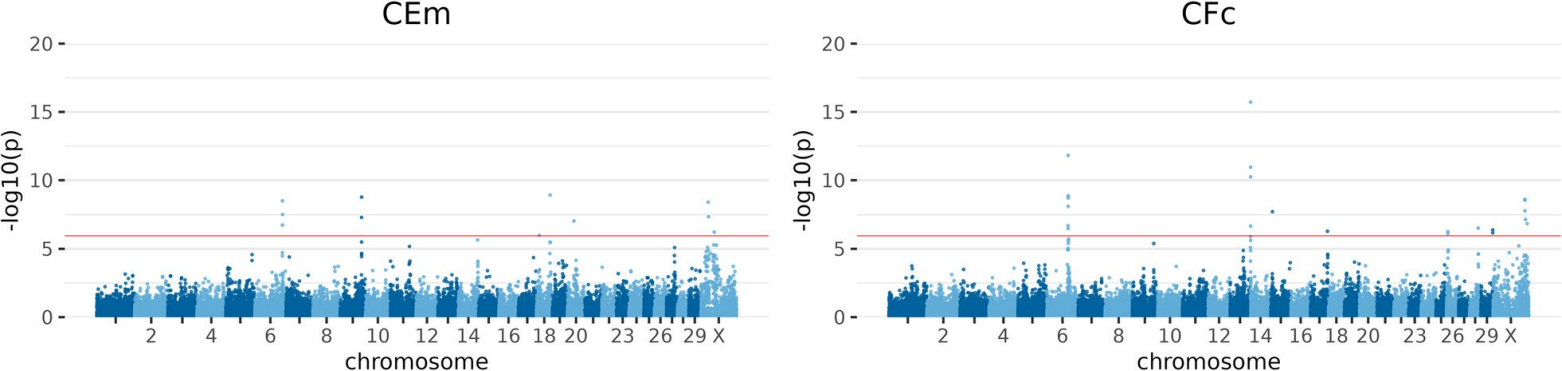
Manhattan plots for exemplary results of GWAS for reproduction traits. Calving ease maternal (CEm) and calving to first insemination (CFc). Negative decadic logarithm of *p-*value of each SNP is shown on the y-axes, and on the x-axes, the 29 autosomes and X chromosome are shown. The red line represents the significance threshold on genome-wide level *p* = 1.11 * 10^−6^

Genome-wide significant signals were identified for the 13 reproduction traits (CEm and CFc in Fig. 3) on BTA5, 6, 7, 9, 12, 14, 15, 17, 18, 20, 21, 23, 26, 28, 29, and BTAX. In total, two to 33 genome-wide significant SNPs were identified per trait. Furthermore, within the context of all chromosomes, BTAX revealed the highest number of significant associations, with a total of 12 distinct traits. Of all autosomes, BTA6 and BTA18 followed, including significant signals for a maximum of seven different traits concurently. The strongest signal for both autosomes was found for BTB-00277427 on BTA6 and ARS-BFGL-NGS-109285 on BTA18.

### Mendelian randomisation

For GSMR analyses, 135 out of 156 trait combinations surpassed the *p*_GSMR_ threshold for significance (*p*_GSMR_ < (0.05/number of SNP_INDEX_)). Following the homogeneity filtering process, the number of identified SNP_GSMR_ as instrumental variables ranged from three to 31. Furthermore, the number of SNPs tested for the 13 reproduction-associated outcome traits (SNP_OUT_) was constant. However, the number of SNPs tested per exposure (SNP_EXP_) varied depending on the trait. An overview of the outcomes is shown in Table 3, while Additional file 4, Table S5 displays detailed results for the number of identified SNP_GSMR_ and the corresponding *p*_GSMR_ value for each combination.

**Table 3:** Overview of GSMR result table for all exposures and outcomes

Exposure	SNP_EXP_	SNP_OUT_	SNP_INDEX_	Significant traits	SNP_GSMR_	Range b_xy_
Conformation						
BCS	101	135	31	12	10–17	− 0.499–0.386
MTY	75	135	30	13	09–18	− 0.400–0.514
OCS	5	135	21	8	04–09	− 0.323–0.262
OBS	68	135	40	12	09–20	− 0.307–0.298
OUS	32	135	30	13	08–15	− 0.526–0.320
OFL	4	135	25	10	04–12	− 0.933–1.335
Metabolism						
KET	8	135	29	13	03–16	− 0.909–1.543
LMV	5	135	26	12	04–14	− 0.822–0.883
MIF	6	135	27	13	10–22	− 1.496–1.244
Production						
FKG	117	135	43	10	10–18	− 0.208–0.415
MKG	110	135	56	10	16–31	− 0.207–0.349
PKG	72	135	41	9	08–14	− 0.231–0.557

Results for different exposure traits from the different complexes tested. SNP_EXP_ represents the number of genome-wide significant (*p* = 1.11 * 10 ^−6^) SNPs for exposure traits, and SNP_OUT_ all genome-wide significant (*p* = 1.11 * 10 ^−6^) SNPs for reproduction traits, respectively. SNP_INDEX_ equals the number of SNPs after the additional clumping filter. Significant traits indicate how many of the 13 tested reproduction traits reached the *p*_GSMR_ significance threshold. At the same time, SNP_GSRM_ and range b_xy_ represent the number of SNPs and effect size for each of the remaining significant combinations, respectively. Trait abbreviations are explained in Table 1

Out of the 39 combinations tested for the three analysed production traits (FKG, MKG, PKG), ten were below the threshold for statistical significance, ranging from eight to 31 SNP_GSMR_ and a b_xy_ between b_xy_ = − 0.231 for PKG as exposure for ZYS (PKG → ZYS) and b_xy_ = 0.557 (PKG → FSc). Additionally, MKG had the highest SNP_INDEX_ (n = 56) within this group and across all production traits, followed by FKG with a SNP_INDEX_ of 43 and PKG with a SNP_INDEX_ of 41.

All combinations except one (LMV → SBd), KET, LMV, and MIF, representing metabolic traits, exceeded the corresponding significance threshold. The combination of KET → CFc had the lowest number of SNP_GSMR_, with three different SNP_GSMR_, while MIF → ZYS had the highest number, with 22 different SNP_GSMR_. The range of b_xy_ for metabolism was higher than all combinations, varying between b_xy_ = − 1.496 (MIF → FSc) and b_xy_ = 1.543 (KET → NGV). The lowest number of SNP_INDEX_ was observed for LMV (n = 26), followed by MIF with 27 different SNP_GSMR._ The highest number was observed for KET, with 29 different SNP_GSMR_.

There were 78 combinations of the six traits and conformation scores tested, of which 68 exceeded the significance threshold. Within this group, we identified four to 20 different SNP_GSMR_, each with a corresponding b_xy_ value. These values ranged from b_xy_ = − 0.933 (OFL → NRh) to b_xy_ = 1.335 (OFL → FSh). The highest number of SNP_INDEX_ was obtained for OBS, including 40 different SNP_GSMR_, while the lowest amount was observed for OCS, with only 21. Additional file 4, Table S5 provides further details on all six traits, and Table 4 displays the results for the detected effect sizes b_xy_.

**Table 4:** b_xy_ results for all combinations of exposure and outcome

Trait	CEd	CEm	CFc	DOc	FSc	FSh	MET	NGV	NRc	NRh	SBd	SBm	ZYS
BCS	0.224 (sign)	0.351 (sign)	− 0.431 (sign)	− 0.499 (sign)	− 0.435 (sign)	− 0.291 (sign)	0.196 (sign)	0.242 (sign)	0.278 (sign)	–	0.226 (sign)	0.318 (sign)	0.386 (sign)
MTY	− 0.310 (sign)	− 0.365 (sign)	0.351 (sign)	0.203 (sign)	0.514 (sign)	0.367 (sign)	− 0.227 (sign)	− 0.293 (sign)	− 0.356 (sign)	− 0.206 (sign)	− 0.299 (sign)	− 0.177 (sign)	− 0.400 (sign)
OCS	–	− 0.315 (sign)	− 0.242 (sign)	–	–	− 0.253 (sign)	0.262 (sign)	0.217 (sign)	− 0.297 (sign)	0.210 (sign)	–	− 0.323 (sign)	–
OBS	− 0.285 (sign)	–	0.298 (sign)	0.153 (sign)	− 0.211 (sign)	0.208 (sign)	− 0.209 (sign)	0.100 (sign)	− 0.307 (sign)	− 0.085 (sign)	− 0.171 (sign)	0.231 (sign)	0.254 (sign)
OUS	− 0.514 (sign)	0.240 (sign)	− 0.321 (sign)	− 0.335 (sign)	− 0.227 (sign)	− 0.316 (sign)	0.222 (sign)	0.282 (sign)	0.320 (sign)	− 0.144 (sign)	− 0.526 (sign)	0.114 (sign)	0.290 (sign)
OFL	–	–	− 0.464 (sign)	*0.743* (sign)	0.581 (sign)	*1.335* (sign)	0.405 (sign)	0.374 (sign)	− 0.225 (sign)	− *0.933* (sign)	0.207 (sign)	–	0.468 (sign)
KET	0.430 (sign)	0.561 (sign)	− 0.225 (sign)	− *0.836* (sign)	− *0.909* (sign)	− 0.547 (sign)	*0.806* (sign)	*1.543* (sign)	0.575 (sign)	*0.786* (sign)	0.389 (sign)	0.541 (sign)	0.617 (sign)
LMV	− 0.409 (sign)	0.585 (sign)	− 0.591 (sign)	− *0.822* (sign)	− *0.723* (sign)	− 0.470 (sign)	*0.857* (sign)	0.395 (sign)	*0.883* (sign)	0.619 (sign)	–	0.539 (sign)	0.678 (sign)
MIF	0.404 (sign)	0.517 (sign)	− *0.977* (sign)	− *1.371* (sign)	− *1.496* (sign)	− 0.645 (sign)	0.497 (sign)	*1.244* (sign)	0.502 (sign)	*0.769* (sign)	0.409 (sign)	0.578 (sign)	*0.789* (sign)
FKG	–	− 0.094 (sign)	0.250 (sign)	0.377 (sign)	0.415 (sign)	0.319 (sign)	− 0.084 (sign)	0.209 (sign)	− 0.177 (sign)	− 0.127 (sign)	–	–	− 0.208 (sign)
MKG	–	0.077 (sign)	0.296 (sign)	0.350 (sign)	0.267 (sign)	0.199 (sign)	− 0.207 (sign)	− 0.161 (sign)	0.103 (sign)	0.048 (sign)	–	–	− 0.120 (sign)
PKG	–	− 0.215 (sign)	0.438 (sign)	0.353 (sign)	0.557 (sign)	0.358 (sign)	–	− 0.175 (sign)	− 0.143 (sign)	− 0.091 (sign)	–	–	− 0.231 (sign)

Exposure traits are listed vertically, and outcome traits are listed horizontally. The exposure traits are grouped by complex (conformation, metabolism, production). Values for b_xy_ greater than |0.700| are shown in italics; non-significant combinations are left empty. Trait abbreviations are explained in Table 1. Detailed results and corresponding *p-*values in Additional file 4, Table S5 and Additional file 5, Figures S5 to S7

The exposures KET, MIF, MTY, and OUS significantly affected all outcomes. Conversely, all tested exposures displayed significant interrelationships with CFc, FSh, NGV, and NRc. The traits related to production had the lowest effect size, ranging from b_xy_ = − 0.231 to 0.557, followed by conformation traits ranging from b_xy_ = − 0.933 to 1.335. The complex that exhibited the largest variation in effect size was associated with metabolic traits, with a range of b_xy_ values from b_xy_ = − 1.496 to  1.244. In terms of conformation, OFL displayed a notably wider range of variation compared to the other conformation and production traits, which had a relatively similar variance (Table 3).

### Downstream analysis

The number of SNP_GSMR_ to be tested for their proximity to known genes per exposure exceeded the range previously presented as SNP_EXP_. This outcome was due to the different individual counts of SNP_GSMR_ for each unique trait combination. Furthermore, for each exposure, we identified both common and trait-specific SNPs. We included any SNP_GSMR_ in the data set associated with at least one of the significant reproduction traits per exposure. Of the four selected exposures, we used 128 different SNP_GSMR_ that exceeded the significance threshold per complex. The results of this selection were 47 genes being identified.

Thirty-two unique SNP_GSMR_ were analysed for BCS, and 12 nearby genes were identified, including *GYS2* on BTA5, *KCNQ1* on BTA29, and *NPFFR2* on BTA6. Similarly, 28 SNP_GSMR_ were identified for KET analysis, enclosing ten genes, including *ST8SIA1* on BTA5 and *NPFFR2* on BTA6. Additionally, for OBS, we obtained 40 different SNP_GSMR_ representing 14 genes, including *LIN28A* on BTA2 and *INSR* on BTA7. Finally, 53 different SNP_GSMR_ were detected for MKG, leading to the identification of 22 genes, including *PAAF1* on BTA15 and *GHR* on BTA20.

**Fig. 4:**
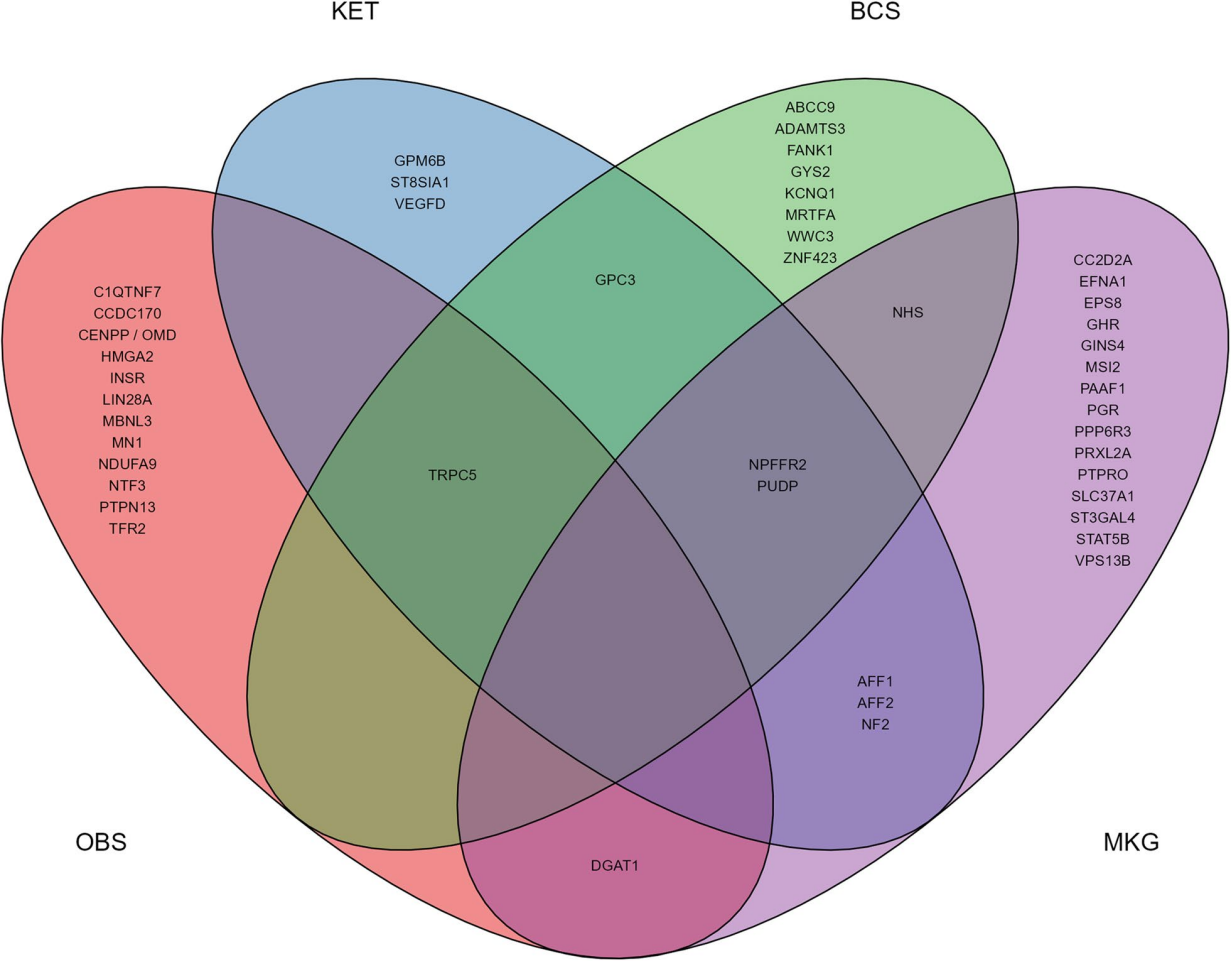
Venn diagram of the selected traits per complex for exposures. Results for the 47 different genes that were identified within the four selected exposure traits as having a significant effect on the outcome. Overall body score (OBS), ketosis (KET), body condition score (BCS) and milk kg (MKG)

Of the four selected exposures, 18 SNP_GSMR_ were present in at least two different exposure traits involving nine genes. These genes included *NPFFR2*, *AFF1* (both BTA6), *DGAT1* (BTA14), *NF2* (BTA17), and on BTAX *GPC3*, *AFF2*, *TRPC5*, *NHS*, and *PUDP*. All identified genes and overlaps between exposures are shown in Fig. 4.

## Discussion

### Genome-wide association studies

We conducted a GWAS on several traits using 235,164 different German Holstein dairy cows as input data for the chosen MR approach. We observed significant genome-wide signals on the autosomes and BTAX for all tested traits, with most hits seen for performance traits and the known lowly heritable functional traits [48]. For instance, in the case of KET, we detected several associated markers on BTA6, including ARS-BFGL-NGS-118182, ARS-BFGL-NGS-17376, and BTB-01654826. Notably, Nayeri et al. [49] also identified ARS-BFGL-NGS-118182 as a marker for KET. Soares et al. [50] described a window on BTA6 ranging from 87,840,175 to 88,893,733 bp for subclinical KET, including all three markers found in this study.

Moreover, several authors identified different SNPs next to the *GC* gene region (86,953,984 – 87,007,062 bp) directly adjacent to BTA6, which are associated with ketosis or other metabolic disorders in Holstein dairy cattle [51–53]. This finding aligns with additional markers found on BTA6 for other metabolic traits (e.g. BTB-00133212 for MIF). According to McCarthy et al. [54], a larger sample size or meta-analysis could increase the statistical power needed to detect significant associations, especially for lower incidence or complex traits. Furthermore, the numerous hits on BTAX support Sanchez et al.’s findings [55] that BTAX harbours great potential to obtain deeper insights into the genetic architecture of complex traits given the high number of markers and associated physiological pathways.

### Variance components

We employed the biREML approach to estimate the direction and relative extent of interrelationships between the traits [39]. Due to computational constraints, we limited the number of animals in the biREML analysis to 48,118. Only those animals that showed expression for both phenotypes tested were included. The biREML analysis revealed weak genetic correlations between exposure and outcome in certain combinations with higher standard errors (see Table 2). This weak genetic correlation was especially noticeable for functional traits like BCS and overall scores.

Furthermore, we used DRPs as phenotypes for our analyses, which were already adjusted based on a mean term [34]. Using DRPs proved advantageous for the GWAS as it increased variance within the partially binary-coded traits. However, it must be noted that pre-correction could introduce bias when estimating variance components or genetic correlations, compared to potentially reduced bias within GWAS [56]. Similarly, it is crucial to avoid biased GWAS results as they can lead to biased MR outcomes [57]. The DRPs were collected at different stages of the routine breeding value estimation. As a result, some traits were corrected for the population mean and, therefore, inverted, for example a lower DRP value for MET implies a higher susceptibility, which is essential when interpreting the GSMR results in relation to the overall b_xy_. biREML was also used to perform independence control within the exposures and outcomes themselves, as this is one of the main rules for MR [12].

To address potential collider bias, Zhu et al. [17] originally implemented the mtCOJO *(*multi-trait-based conditional and joint analysis) method. However, in this study, we did not attain the 200,000 markers required for LD correction [58, 59]. As previously reported in the literature (e.g. [60]), we identified strong dependencies between the PKG, MKG, and FKG exposure traits and between individual traits from the metabolic and conformational domains. However, the analysis could not quantify three combinations: FSc and FSh versus NRc and BCS versus MTY. After removing the residual covariance, the estimation model revealed a strong genetic correlation among trait combinations (FSh‑NRc r_G_ = − 0.994 ± 0.002, FSc‑NRc r_G_ = − 0.985 ± 0.002, BCS‑MTY r_G_ = − 0.988 ± 0.001).

Based on the reliability of our GSMR results, biREML had the potential to determine the direction and quantification of causal associations between two traits. When both methods were applied, the same DRPs and genotype data (except for BTAX) were subjected to the same potential limitations or sources of bias mentioned above (e.g. correction of the DRPs). The joint use of GSMR and biREML data enabled an investigation into the relationship between traits based on SNPs from biREML and IVs identified from GSMR. The results for each exposure trait were plotted to facilitate a comparison of the two approaches, and the overall correlation between the obtained r_G_ from biREML and b_xy_ of GSMR results was estimated (Additional file 5, Figures S5 to S7).

Results for the linear overall scores we obtained from both methods exhibited minimal correlation without statistical significance (e.g. OFL: r_G_ and b_xy_ r = − 0.01, *p* = 0.961; OCS: r_G_ and b_xy_ r = − 0.14, *p* = 0.630). Nevertheless, there were high correlations between the individual direct traits (e.g. PKG: r_G_ and b_xy_ r = 0.92, *p* *<* 0.001; KET: r_G_ and b_xy_ r = 0.93, *p* *<* 0.001), which emphasise the informative value and suitability of our approach. The potential limitations resulting in non-significant estimates or low correlations between methods could be attributed to the definition of the trait itself [61] or the independence of the individual traits used as instrumental variables [12].

Moreover, the linear overall scores do not directly quantify the traits; rather, they are extrapolated through other traits that are directly measured and subsequently indexed. This indirect quantification is evident when the results are compared between OBS (r_G_ and b_xy_ r = 0.46, *p* = 0.111) and BCS (r_G_ and b_xy_ r = 0.89, *p* *<* 0.001) (Additional file 5, Figure S5). Both traits are part of the complex conformation. BCS is scored directly and used as an independent trait, whereas OBS is indexed and considers additional factors such as stature, body depth, and chest width.

### Mendelian randomisation

Zhu et al.’s GSMR approach [17] has already been applied to smaller cattle cohorts [62, 63]. This study observed significant effects for all tested exposure traits, with at least eight distinct reproduction-associated traits as outcomes. We identified and removed potential (horizontal) pleiotropic outliers following the HEIDI-outlier approach [26] for all these combinations. Notably, vertical pleiotropy does not invalidate the instrumental variable assumptions for MR, whereas horizontal pleiotropy would violate them [12].

In general, three major assumptions must be fulfilled by the IVs [24, 25]: (1) the IV is associated with the exposure, (2) the IV does not affect the outcome except potentially via the exposure, and (3) the IV is not associated with the outcome due to confounding pathways. We fulfilled these aspects by using a strict GWAS *p-*value significance threshold after Bonferroni correction, which is considered putatively overly conservative [64] but helps to avoid potential bias.

We also applied a strict clumping filter (*r*^*2*^ = 0.05) to remove SNPs in LD blocks with the most significant SNPs per trait. The application reduced the correlation between remaining SNPs while retaining those with the strongest trait-specific statistical evidence [65]. This approach was also relevant for confounding [58] and is supported by the assumption and testing for constant b_xy_ under the hypothesis of direct trait-mediated effects. Thus, there is no heterogeneity in the variant-specific estimates [12, 17]. Although previous studies have described general relationships (e.g. [52]), the main objective was identifying the potential IVs responsible for such effects within a large, somewhat undistorted dataset.

One potential limitation of the GSMR approach is the unavailability of exposure traits and their definitions. In contrast to human studies, where self-determined behaviours such as smoking [22], alcohol consumption [28], or physical activities [29] are frequently used as exposures, comparable characteristics are elusive or unattainable in dairy cattle husbandry. However, negative energy balance could theoretically be used as an estimation characteristic instead of BCS as it does not directly display the breakdown of energy intake into allocation or acquisition over time [66], nor does it show clear interdependency with the investigated reproduction parameters [67]. In addition, measuring devices such as pedometers could be used to collect data on activity behaviour for exposure. However, it is worth noting that activity behaviour can be influenced by physiological processes that affect both the reproductive process [68] and diseases of the movement apparatus, such as lameness [69].

Furthermore, individual factors, such as management conditions, housing structure, herd hierarchy, or herd density, may serve as potential stratification factors for this [70], as opposed to human studies that lack such stratifications. It is also important to note that not all traits have linear relationships, which is not accounted for in the linear approximation of GSMR [17].

For instance, human studies demonstrated a U-shaped relationship between body mass index and mortality [26]. These studies align with the findings of Roche et al. [71], who reported negative impacts on performance, reproduction, and metabolism in dairy cows with high and low BCS scores. However, the selected method enables the quantification and chromosomal localisation of the IVs as drivers of the observed interrelationship. As a result, we can more precisely understand the genetic architecture and distinguish between horizontal and vertical pleiotropic effects.

Regarding the breeding value estimation, the results can be employed to account for known and novel interactions between traits. Moreover, achieving more accurate chromosomal localisation can enhance fine mapping or aid in choosing markers that offer greater informational value. Subsequently, the GSMR and variance component results could improve our understanding of the relationship between complex traits and facilitate further consideration of different traits and their associated weightings within the breeding schemes. A more accurate indexing of different traits could be achieved by better understanding their causal relationships.

### Gene associations

In addition to the statistical evidence, we selected four traits to evaluate the possible physiological backgrounds shown by the IVs that might shed light on the causal associations found. We considered 47 different genes (38 on autosomes and 9 on BTAX) obtained from 128 different putative causal SNP_GSMR_ through database research and subsequent literature review. Additional file 6, and Table S6 provide a more detailed overview of the results for all 128 identified SNP_GSMR_ and their corresponding outcome traits.

As an illustration, the gene *GYS2* (Glycogen Synthase 2) was identified through ARS-BFGL-NGS-103973 on BTA5 at 89,065,689 bp for BCS and has significant putative effects on 11 reproduction traits. Tribout et al. [72] identified *GYS2* as associated with protein yield, while Wathes et al. [73] demonstrated up-regulated *GYS2* expression in metabolically imbalanced Holstein Friesian dairy cows. In humans, *GYS2* has been linked to polycystic ovary syndrome and obesity-related conditions [74]. These results directly link the observed causal connection between BCS conditions and reproductive performance. Furthermore, Dean [75] highlighted the importance of glycogen balance in mammals during early pregnancy and the contribution of *GYS2* to endometrial glycogen levels.

Meanwhile, the gene *NPFFR2* (neuropeptide FF receptor 2) located on BTA6 was identified through the same causal SNP (BTA-68275-no-rs) for three exposures: MKG, KET, and BCS. It was significantly linked to 2, 8, and 12 different reproductive traits, respectively. Examples of known and previously described traits affected by *NPFFR2* in cattle include dairy form, productive life [76], milk, fat and protein yield [77], somatic cell score [76, 78], longevity [79], and clinical mastitis [80]. *NPFFR2* is a functional G protein-coupled receptor involved in regulating the opioid system, cardiovascular function and neuroendocrine function [81]. Endogenous opioids interact with gonadotropin secretion in various mammalian species [82, 83], emphasising the link with reproductive traits.

Likewise, the gene *GHR* (growth hormone receptor, on BTA20 at 31,933,394 bp) identified for MKG showed a significant association with four different reproductive traits. The role of *GHR* in beef and dairy cattle has been widely discussed in the literature, and various associations have already been described, including growth, beef performance (e.g. [84]), and dairy performance (e.g. [85]). Waters et al. [85] identified a potential biomarker for yield evaluation in a *GHR*-related SNP (on BTA20 at 34,153,345 bp) within the untranslated medial exon. They also described the associations with lactation persistency [86], feed efficiency [87], and energy balance [88] as consistent with the suggested interrelationship discovered.

For BCS on BTA29, *KCNQ1* (potassium voltage-gated channel subfamily Q member 1) was found to have a significant causal association with 11 different reproduction traits. Associations for cattle, including luteinising hormone [89], udder depth, and somatic cell score [72], have been described. Lafontaine and Sirard [90] highlighted the crucial role of DNA methylation of *KCNQ1* in bovine follicles, while Chen et al. [91] demonstrated a link between *KCNQ1* and the large offspring syndrome in bovine embryos. In a human study, Gómez-Úriz et al. [92] further highlighted an interaction between obesity and ischaemic stroke with *KCNQ1* methylation.

BTAX, the second-largest chromosome in the bovine genome [93], represents more than 40% of the SNPs significantly identified in this study but less than 20% of the genes identified. Currently, most GWAS studies in cattle, such as Sahana et al. [80], exclude BTAX. However, Su et al. [94] demonstrated that considering markers on BTAX could improve the accuracy of genomic prediction. Moreover, Sanchez et al. [55] showed that X-linked genes impact various traits. In our study, three out of the nine genes we identified, namely *GPC3* (glypican 3), *MBNL3* (muscleblind-like splicing regulator 3), and *TRPC5* (transient receptor potential cation channel subfamily C member 5), have previously been linked to dairy traits and stature [55]. Additionally, we found that the *AFF2* gene (ALF transcription elongation factor 2) we identified on BTAX for KET and MKG was also reported in a recent study, associating it with milk urea nitrogen [95].

Although our study identified various SNPs, genes, and physiological processes that may be relevant to putative causal associations, we want to address the results of Waters et al. [85]. Using *GHR* as an example, they demonstrated the impact that a marker’s position could have on the detected effects. Increasing marker density through sequencing or imputation [96] may enhance the results of GWAS and the detection of effects, thereby facilitating subsequent analyses. However, it is important to note that the limited accuracy of genotype data for GWAS may introduce bias in subsequent analyses [57]. Simulations have demonstrated the effect of the chosen method on the accuracy of genotype imputation [97]. Moreover, accurately estimating the genetic architecture of complex traits requires a large number of individuals [98]. Therefore, a large number of animals and a sufficient density of markers are also required to estimate causal associations successfully between these traits.

## Conclusion

In conclusion, this study estimated the GWAS summary statistics for 25 traits involving 235,164 individuals. Genome-wide significant SNPs for all traits were identified. Furthermore, we estimated variance components between these traits and analysed their causal associations. The GSMR approach detected significant causal association effects on reproduction traits. Subsequent gene association screening confirmed physiologically plausible effects for four traits related to conformation, metabolism, and production.

Moreover, we identified novel associations, providing leads for further analysis. A large sample size ensured reliable results, even for complex traits with strict thresholds. These findings can enhance our understanding of the genetic structure of these traits and their interrelationship beyond genetic correlation, which can be considered in future breeding strategies to preserve health and production simultaneously.

## Supplementary Information

Additional file 1. Tables S1 to S2. Genetic correlation estimates. Table S1 displays the SNP-based genetic correlation (r_G_) estimates among exposure traits. Table S2 displays the SNP-based genetic correlation (r_G_) estimates among outcome traits. Negative values are displayed in red, and r_G_ > |0.200| is in bold. The standard error (SE) for each combination is displayed in parentheses. The red square corresponds to the failed combination.

Additional file 2. Tables S3 to S4. GWAS results. Results for genome-wide association studies for 12 exposure (complex of conformation, metabolism and production) and 13 outcome traits (all reproduction) surpassing the genome-wide significance threshold after Bonferroni-correction (*p* = 1.11 * 10^-6^ [(0.05/44,994), - log_10_ (P) ≈ 5.95]. For each SNP per trait surpassing the significance threshold, the chromosome (Chr), marker localisation in base pairs (bp) and marker name (SNP) are given. Further, the respective alleles and frequencies (A1, A2, Freq), the signed Z-scores (Zscore) and the *p*-value (*p*).

Additional file 3. Figures S1 to S4. Manhattan plots. Manhattan plots for the genome-wide association studies for the remaining exposure (complex of conformation, metabolism and production) and outcome traits (all reproduction) not shown within the manuscript. Negative decadic logarithm of *p-*value of each SNP is shown on the y-axes, and on the x-axes, the 29 autosomes and X chromosome are shown. The red line represents the significance threshold on genome-wide level *p* = 1.11 * 10^-6^*.* The genomic inflation factor was fixed to 1 due to the genomic correction flag used by METAL for merging the cohorts together.

Additional file 4. Table S5. GSMR result table. Result tables for the GSMR analysis including all 156 combinations (135 significant, 21 non-significant). Shown are exposure traits of all complexes against the individual outcome traits (reproduction), estimated effect per combination (b_xy_) including standard error (se) and *p*-value for the test statistic (p_GSMR_). The number of SNPs after filtering (nsnp) and the number of SNPs tested within the run (SNP_INDEX_). Last, the threshold for significance is given (*p*_GSMR_ < (0.05/SNP_INDEX_ = p_thresh_) and if the specific combination surpassed this (significant/non-significant). Traits and abbreviations are listed separately and given in the main manuscript, Table 1.

Additional file 5. Figure S5 to S7. Correlation between variance components and GSMR. For all putative exposure traits, sorted by the three complexes conformation, metabolism and production, results for the variance component estimation (r_G_) and the GSMR result (b_xy_) are plotted against each other for all exposure (Trait). Each data point is labelled with the corresponding abbreviation of the reproduction trait. Number of significant outcomes (No. signif. outcomes) is stated, and the non-significant traits are named (n.s. outcomes). Further, the range of SNP_GSMR_ identified for all corresponding outcomes is given (range SNP_GSMR_, details see Additional file 4, Table S5). The estimated correlation between variance component estimation and the GSMR results (r(r_G_, _bxy_)) is stated together with the corresponding *p-*value (p-value) of correlation estimation.

Additional file 6. Table S6. Downstream analysis results top traits. Four traits (BCS, OBS, KET, MKG) representing the three different complexes (conformation, metabolism and production) were further selected and processed. The table displays all the identified SNPs within the four exposure traits in at least one combination with an outcome. Chromosome (Chr) and marker localisation in base pairs (bp) are given, as well as individual identifiers (rsNumbers) and marker name (SNP). Next, the 13 different outcome traits tested (CEd, CEm, CFc, DOc, FSc, FSh, MET, NGV, NRc, NRh, SBd, SBm and ZYS) are listed. A black cross indicates a significant identification within the GSMR approach, a red cross non-significant result. Empty fields indicate no association at all was identified. The putative causal exposure trait per SNP (Exposure) is given and reveals which of the four tested was relevant here. Last, the identified genes, including the gene symbol (Genes) within a 1000 bp up and downstream of the identified marker.

## Data Availability

The SNP chip genotype data and phenotypes cannot be shared publicly, as they are property of the German Holstein breeding organisations organised in the umbrella federation BRS (Bundesverband Rind und Schwein e.V., Bonn, Germany), deregressed proofs are computed by the national computing center VIT (Vereinigte Informationssysteme Tierhaltung w.V., Verden) in Germany. Summary statistics for genome-wide significant GWAS and GSMR results are provided in the Additional files 1 to 6. Further summary statistics can be provided upon reasonable request.
