# Supplementary material for: Mendelian randomisation to uncover causal associations between conformation, metabolism, and production as potential exposure to reproduction in German Holstein dairy cattle
Source: Genet Sel Evol. 2025 Feb 25;57:7. doi: 10.1186/s12711-025-00950-w (PMC11863791; doi:10.1186/s12711-025-00950-w)
Supplement: Supplementary file 3 — Additional file 3. Figures S1 to S4. Manhattan plots. Manhattan plots for the genome-wide association studies for the remaining exposure (complex of conformation, metabolism and production) and outcome traits (all reproduction) not shown within the manuscript. Negative decadic logarithm of p-value of each SNP is shown on the y-axes, and on the x-axes, the 29 autosomes and X chromosome are shown. The red line represents the significance threshold on genome-wide level p = 1.11 * 10-6. The genomic inflation factor was fixed to 1 due to the genomic correction flag used by METAL for merging the cohorts together. [file 12711_2025_950_MOESM3_ESM.pdf]

Figure S1, Conformation

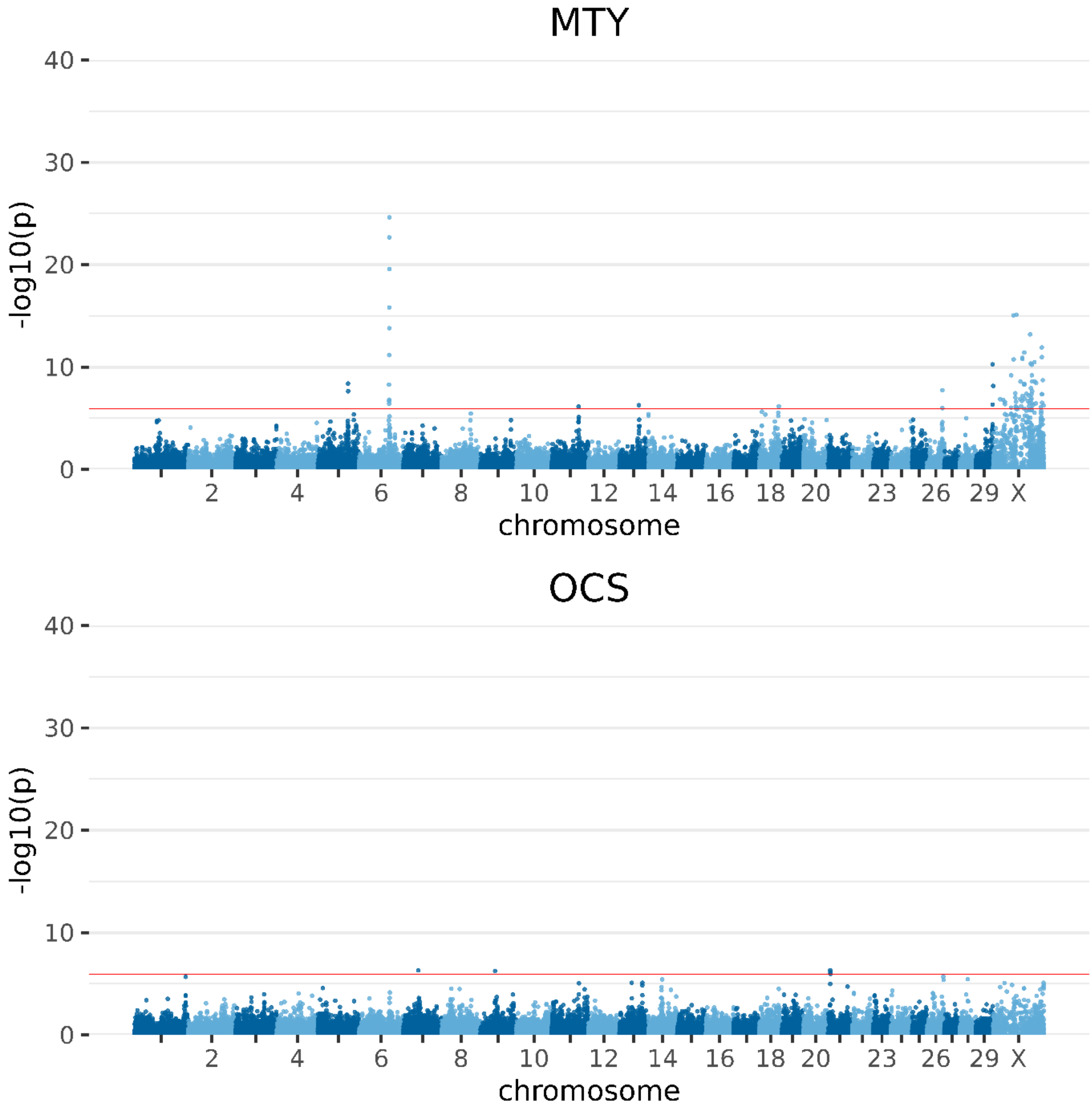

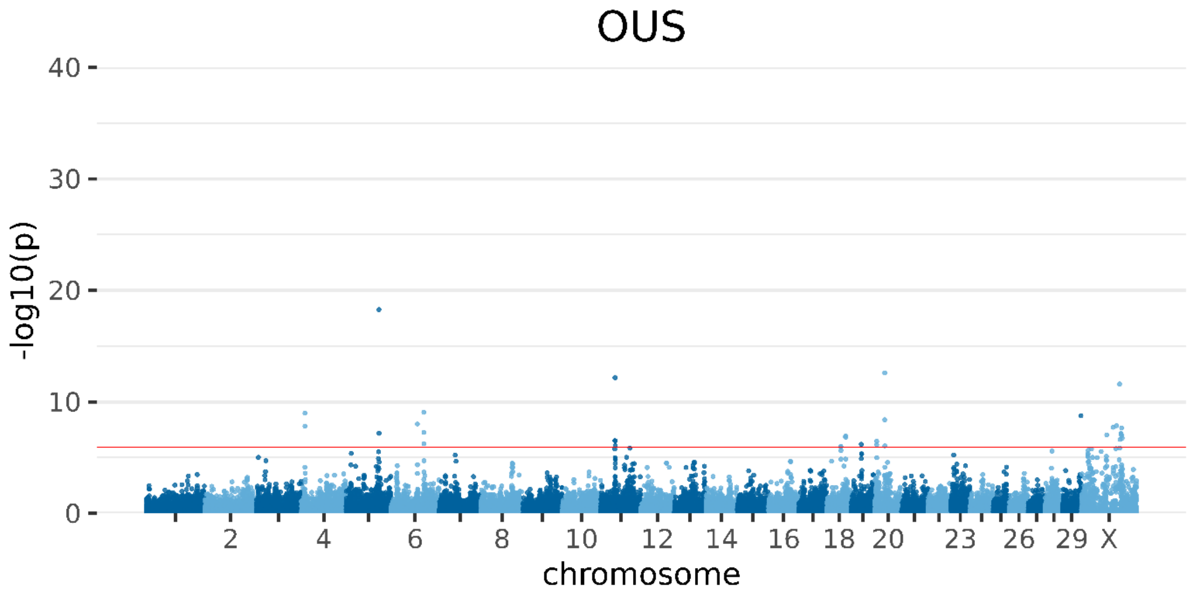
OUS
40
30
20
10
0
-log10(p)
2
4
6
8
10
12
14
16
18
20
23
26
29
X
chromosome

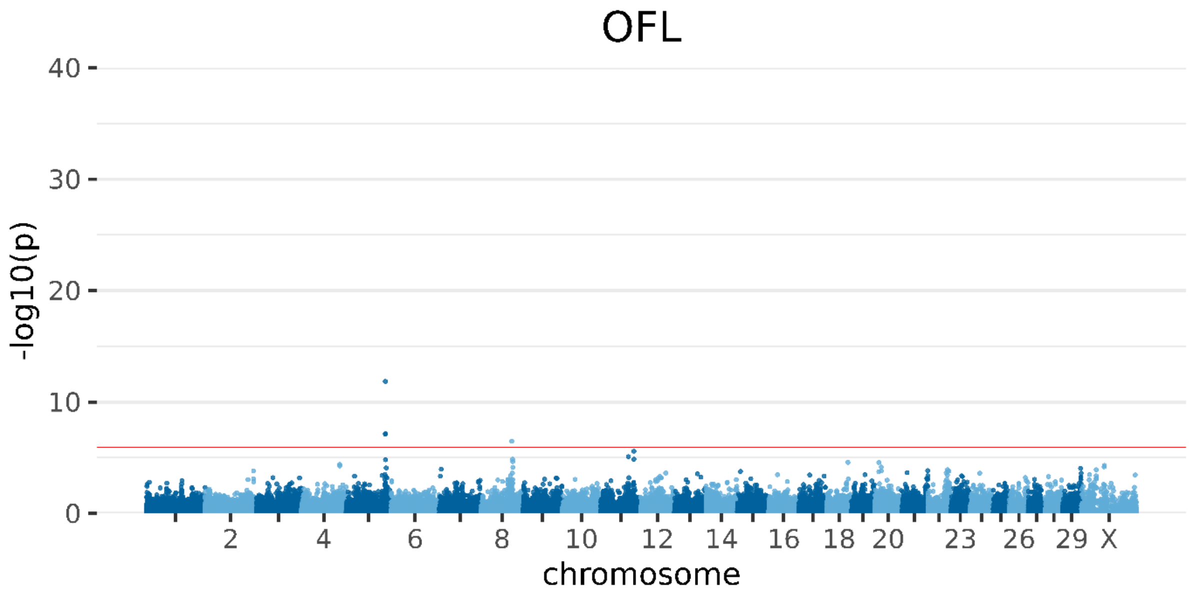
OFL
40
30
20
10
0
-log10(p)
2
4
6
8
10
12
14
16
18
20
23
26
29
X
chromosome

Figure S2, Metabolism

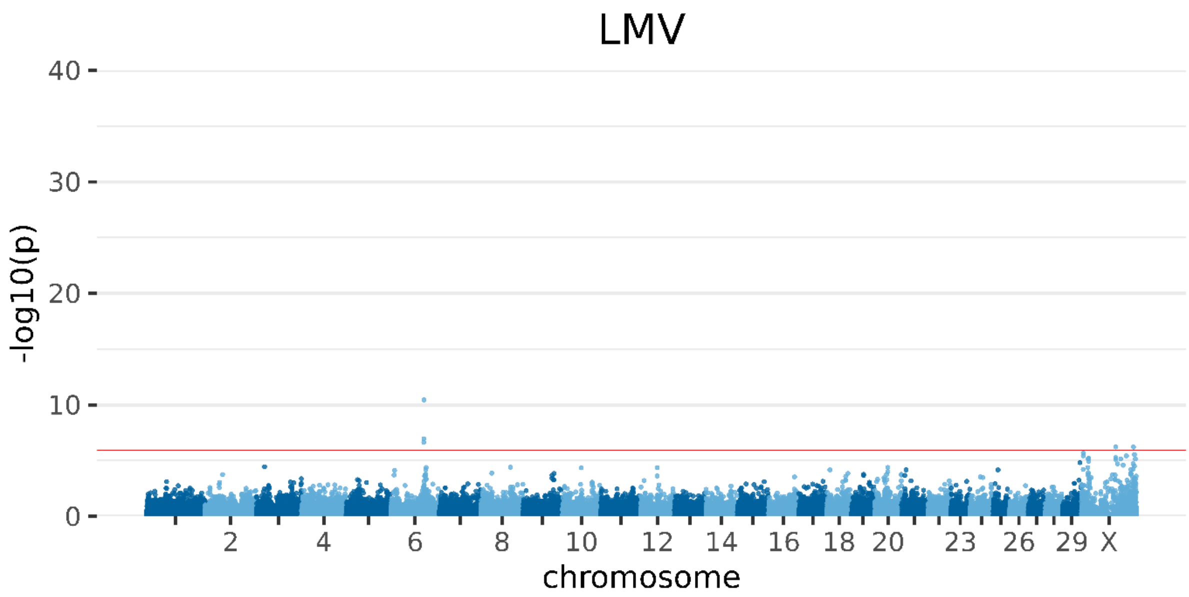

Figure S3, Production

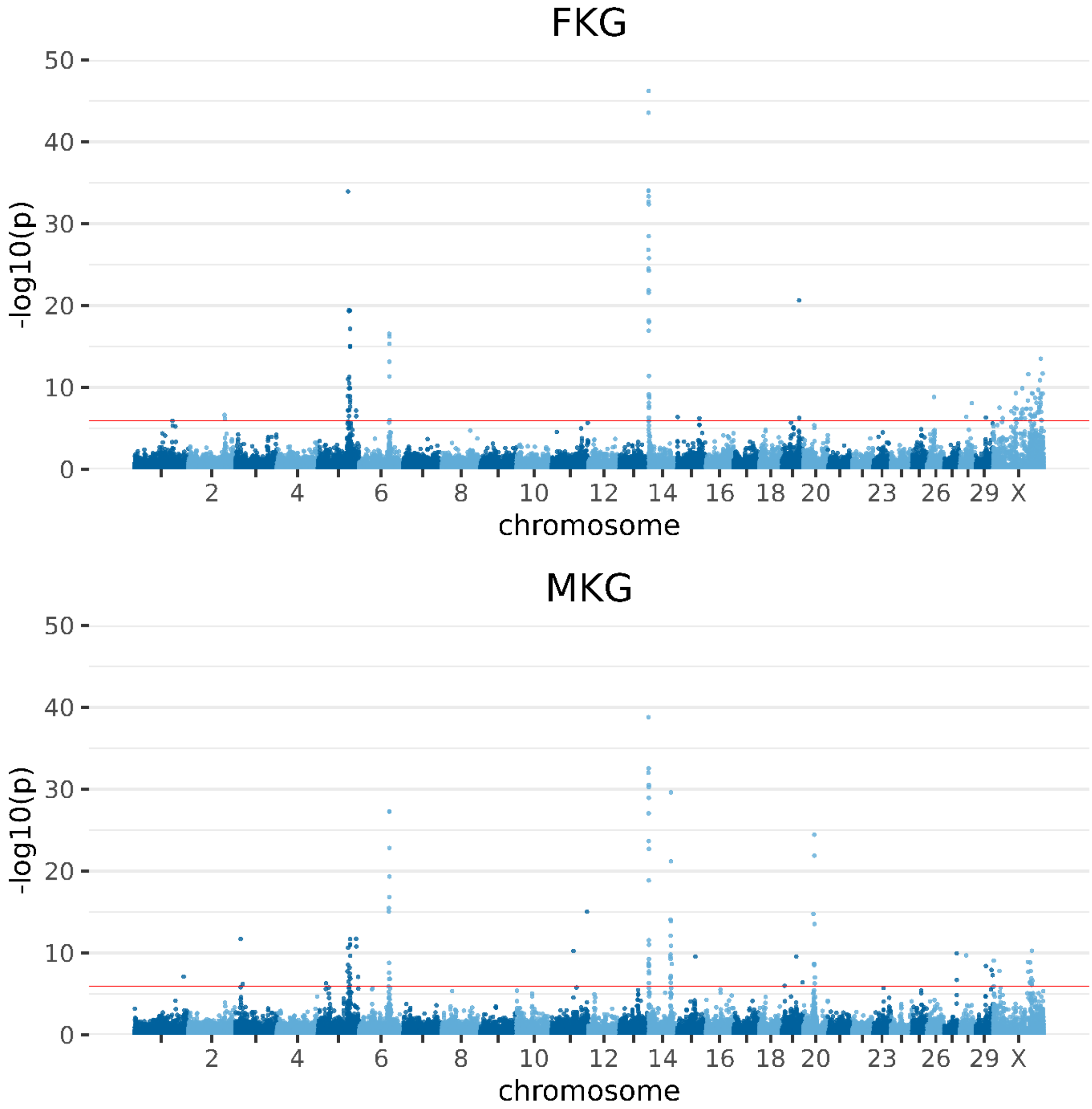

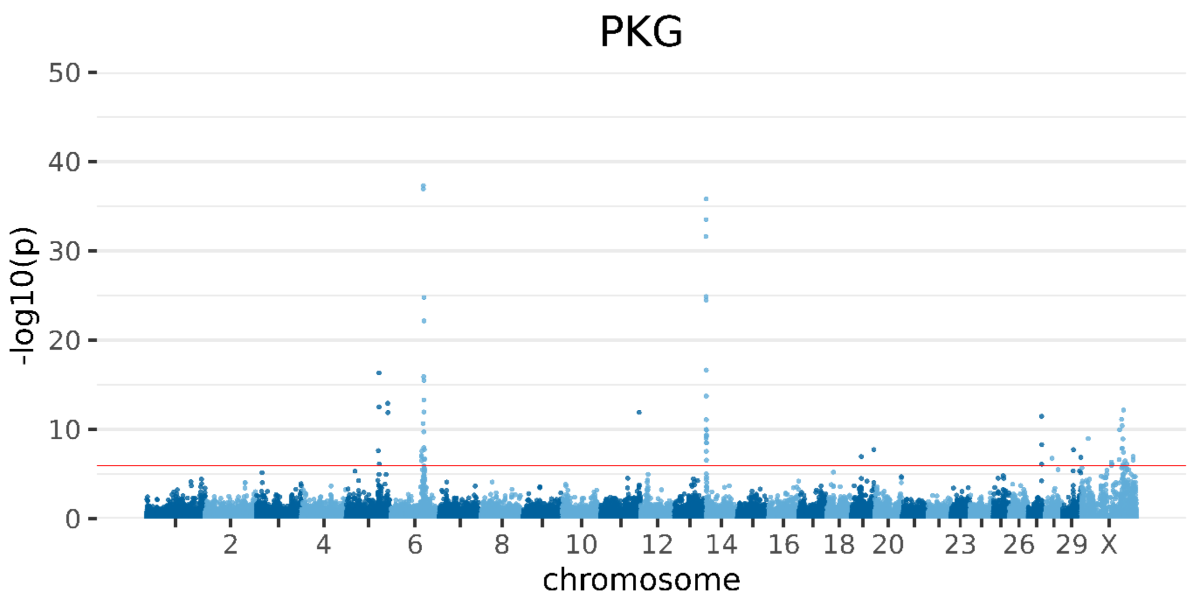
PKG
-log10(p)
50
40
30
20
10
0
2
4
6
8
10
12
14
16
18
20
23
26
29
X
chromosome

Figure S4, Reproduction

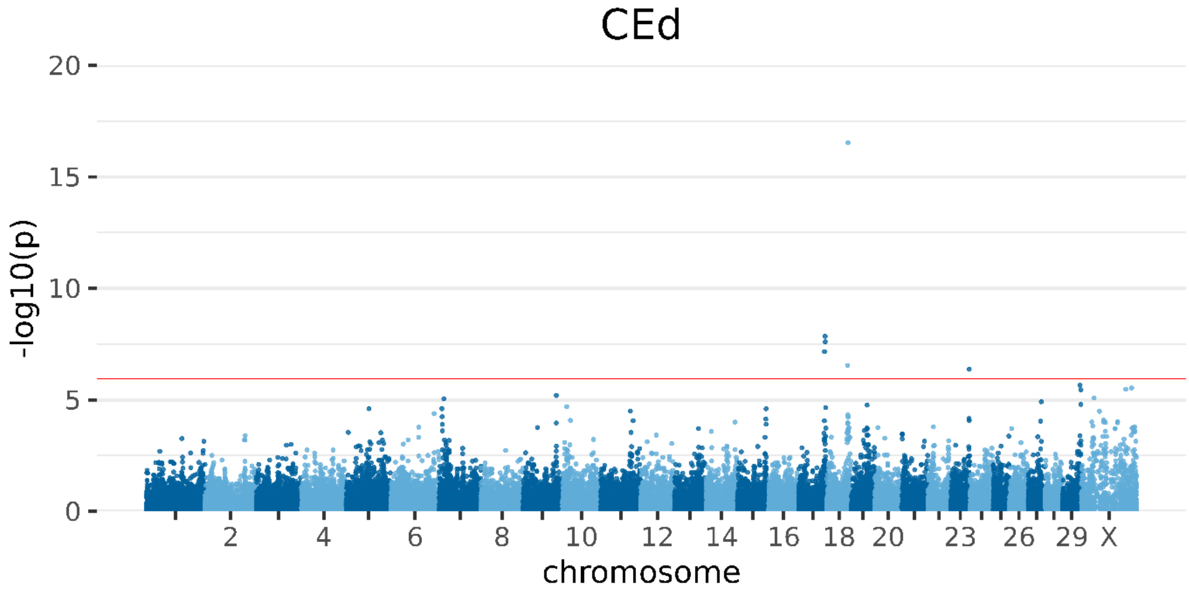

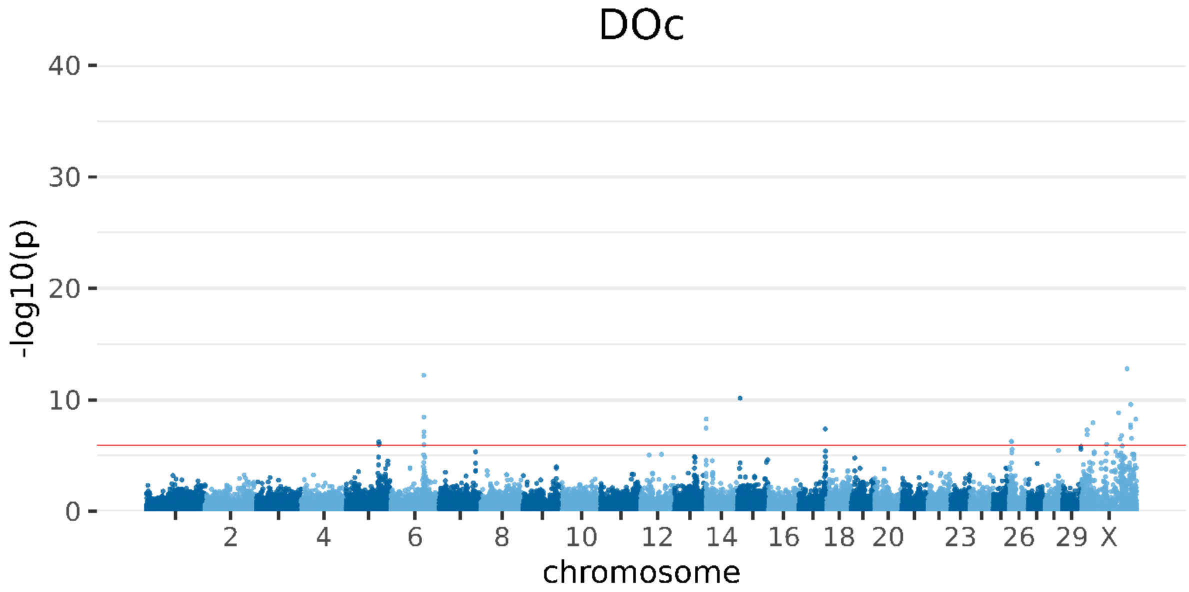

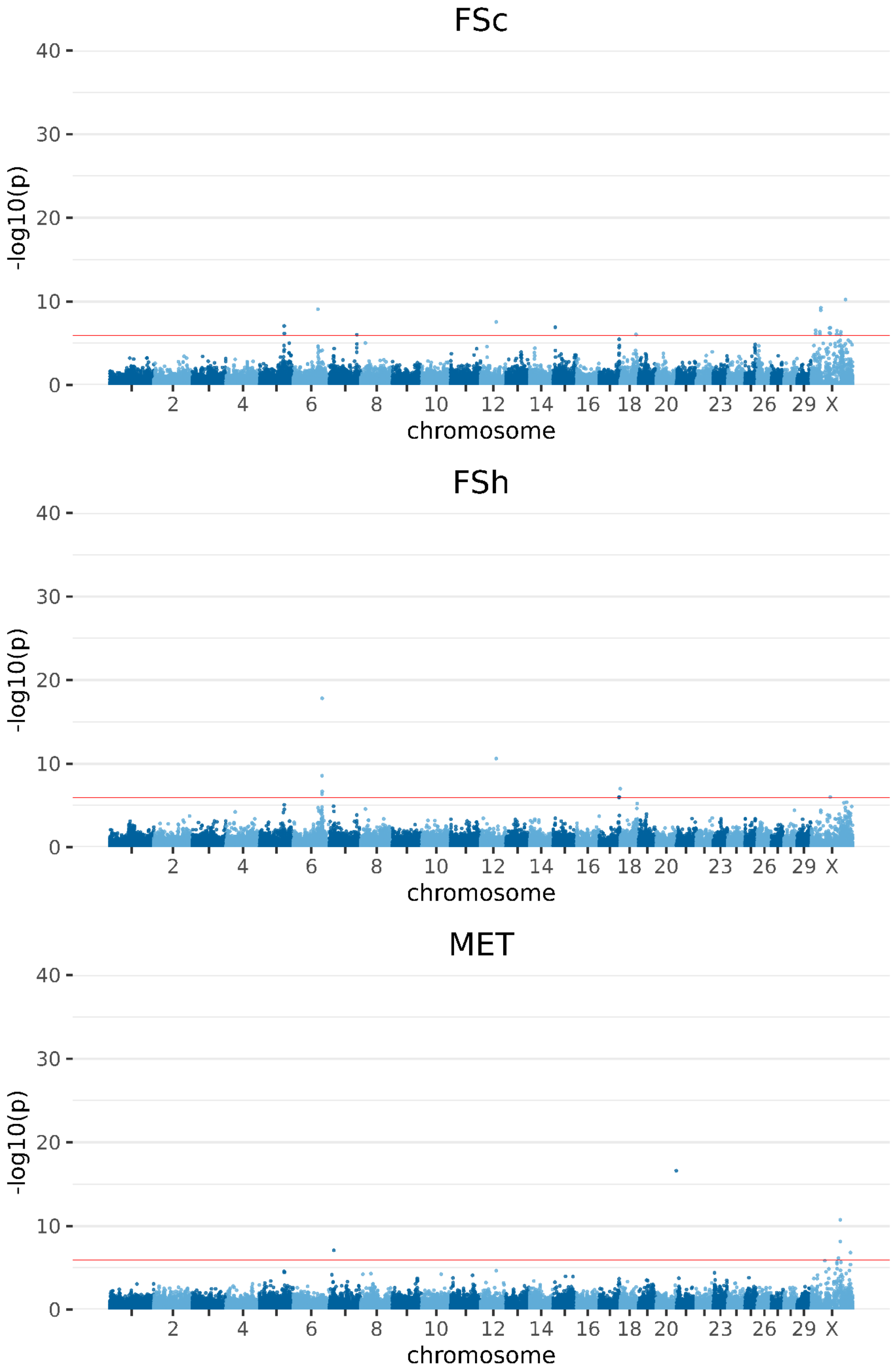
FSc
40
30
20
10
0
-log10(p)
2 4 6 8 10 12 14 16 18 20 23 26 29 X
chromosome
FSh
40
30
20
10
0
-log10(p)
2 4 6 8 10 12 14 16 18 20 23 26 29 X
chromosome
MET
40
30
20
10
0
-log10(p)
2 4 6 8 10 12 14 16 18 20 23 26 29 X
chromosome

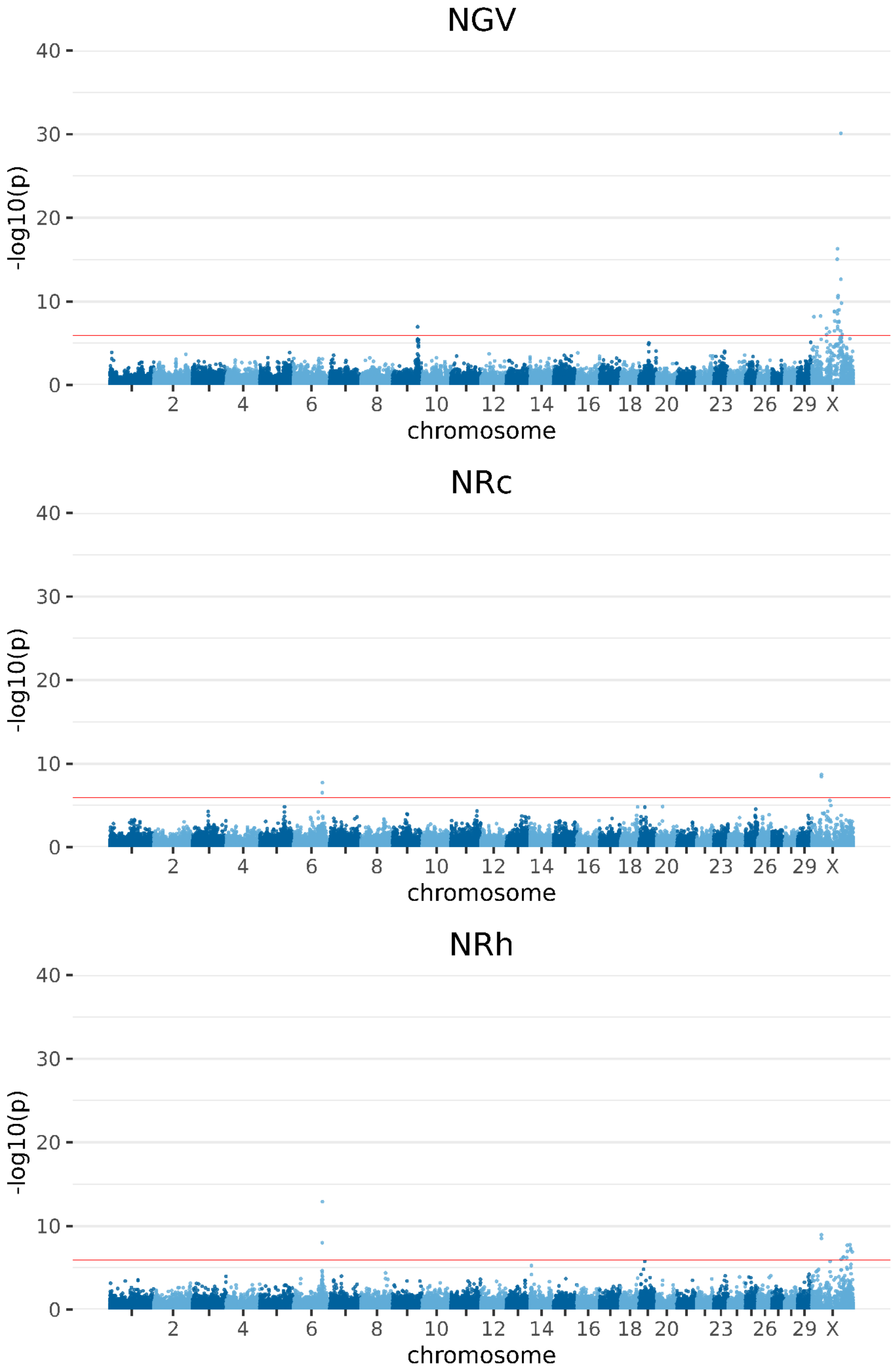

NGV
NRc
NRh
-log10(p)
chromosome
0
10
20
30
40
2
4
6
8
10
12
14
16
18
20
23
26
29
X

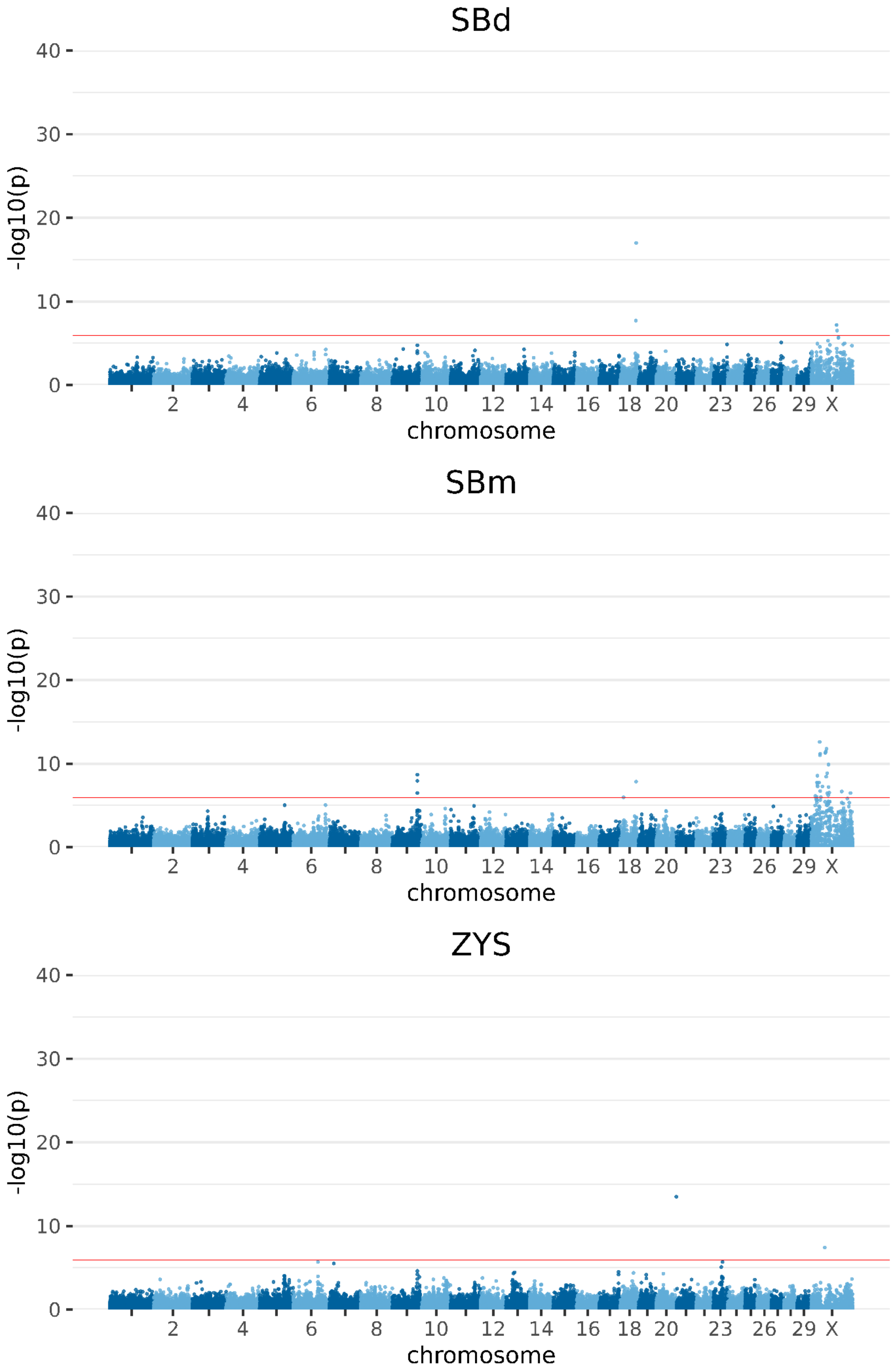

SBd
-log10(p)
40
30
20
10
0
2
4
6
8
10
12
14
16
18
20
23
26
29
X
chromosome
SBm
-log10(p)
40
30
20
10
0
2
4
6
8
10
12
14
16
18
20
23
26
29
X
chromosome
ZYS
-log10(p)
40
30
20
10
0
2
4
6
8
10
12
14
16
18
20
23
26
29
X
chromosome
